# The DRS–AIMP2–EPRS subcomplex acts as a pivot in the multi-tRNA synthetase complex

**DOI:** 10.1107/S2052252519010790

**Published:** 2019-08-24

**Authors:** Hyunggu Hahn, Sang Ho Park, Hyun-Jung Kim, Sunghoon Kim, Byung Woo Han

**Affiliations:** a Research Institute of Pharmaceutical Sciences, College of Pharmacy, Seoul National University, Seoul 08826, Republic of Korea; b College of Pharmacy, Chung-Ang University, Seoul 06974, Republic of Korea; c Medicinal Bioconvergence Research Center, Department of Molecular Medicine and Biopharmaceutical Sciences, Seoul National University, Seoul 08826, Republic of Korea

**Keywords:** aspartyl-tRNA synthetase, aminoacyl-tRNA synthetase complex-interacting multifunctional protein 2, glutamyl-prolyl-tRNA synthetase, multi-tRNA synthetase complex

## Abstract

Aminoacyl-tRNA synthetases (ARSs) play essential roles in protein biosynthesis as well as in other cellular processes, often using evolutionarily acquired domains. For possible cooperativity and synergistic effects, nine ARSs assemble into the multi-tRNA synthetase complex (MSC) with three scaffold proteins: aminoacyl-tRNA synthetase complex-interacting multifunctional proteins 1, 2 and 3 (AIMP1, AIMP2 and AIMP3). X-ray crystallographic methods were implemented in order to determine the structure of a ternary subcomplex of the MSC comprising aspartyl-tRNA synthetase (DRS) and two glutathione *S*-transferase (GST) domains from AIMP2 and glutamyl-prolyl-tRNA synthetase (AIMP2_GST_ and EPRS_GST_, respectively). While AIMP2_GST_ and EPRS_GST_ interact via conventional GST heterodimerization, DRS strongly interacts with AIMP2_GST_ via hydrogen bonds between the α7–β9 loop of DRS and the β2–α2 loop of AIMP2_GST_, where Ser156 of AIMP2_GST_ is essential for the assembly. Structural analyses of DRS–AIMP2_GST_–EPRS_GST_ reveal its pivotal architecture in the MSC and provide valuable insights into the overall assembly and conditionally required disassembly of the MSC.

## Abstract 2

The ternary complex structure of human aspartyl-tRNA synthetase (DRS) and two glutathione *S*-transferase domains from aminoacyl-tRNA synthase complex-interacting multifunctional protein 2 and glutamyl-prolyl-tRNA synthetase (AIMP2_GST_ and EPRS_GST_, respectively) was determined at 3.6 Å resolution, providing insight into the pivotal role of the ternary complex within the multi-tRNA synthetase complex.

## 1. Introduction   

Aminoacyl-tRNA synthetases (ARSs) play an essential role in protein biosynthesis through the attachment of amino acids to their cognate tRNAs, which is the first step in the translation process (Berg & Offengand, 1958 ▸). In addition to their primary role in protein synthesis, ARSs of higher eukaryotes additionally play roles in essential nontranslational functions through the acquisition of additional motifs or domains, or by generating alternative splicing variants which exhibit different physiological effects (Guo *et al.*, 2010 ▸; Lo *et al.*, 2014 ▸).

Nine of the 20 cytosolic ARSs interact with three scaffold proteins: amino­acyl-tRNA synthetase complex-interacting multifunctional proteins 1, 2 and 3 (AIMP1, AIMP2 and AIMP3). Together, they assemble into the multi-tRNA synthetase complex (MSC), with a molecular weight of ∼1.5 MDa (Rho *et al.*, 1999 ▸; Robinson *et al.*, 2000 ▸). The formation of the MSC has been known to expedite protein synthesis through sequestering and stabilizing the components, which are otherwise rapidly degraded (Han *et al.*, 2006 ▸). Thus, the MSC provides a constant flow of charged tRNA for the translational machinery (Negrutskii & Deutscher, 1991 ▸; Kyriacou & Deutscher, 2008 ▸). Conversely, nontranslational functions of ARSs were often displayed following dissociation from the MSC in response to various stress signals (Lee *et al.*, 2004 ▸). The spectrum of such downstream signal pathways ranges from proliferation (Ko *et al.*, 2000 ▸; Han *et al.*, 2012 ▸) and tumorigenesis (Choi *et al.*, 2011 ▸) to tumor suppression (Han *et al.*, 2008 ▸) and apoptosis (Ko *et al.*, 2001 ▸).

Among the three scaffold proteins, AIMP2 possesses the most binding sites for MSC components. The N-terminal region of AIMP2 binds the homodimeric interface of lysyl-tRNA synthetase (KRS; Ofir-Birin *et al.*, 2013 ▸), while the adjacent amphipathic α-helix region makes coiled-coil inter­actions with a stable subcomplex composed of arginyl-tRNA synthetase (RRS), glutaminyl-tRNA synthetase (QRS) and AIMP1 (the RQA1 subcomplex; Ahn *et al.*, 2003 ▸). The C-terminal region of AIMP2 contains a glutathione *S*-transferase (GST)-homology domain which structurally facilitates a heterotetrameric GST complex with three different GST-homology domains from glutamyl-prolyl-tRNA synthetase (EPRS), AIMP3 and methionyl-tRNA synthetase (MRS) (the A2EA3M subcomplex; Cho *et al.*, 2015 ▸).

Aspartyl-tRNA synthetase (DRS) is another known binding partner of AIMP2 in the MSC (Kim *et al.*, 2002 ▸). We previously reported the crystal structure and a potential nontranslational role of DRS released from the MSC (Kim *et al.*, 2013 ▸). However, it remains unclear whether there is a direct interaction between AIMP2 and DRS, which prohibits a systematic understanding of MSC assembly and dis­assembly. Hence, we tried to gain insights into the dissociation process by elucidating the protein–protein interaction network within the MSC.

Here, we report the crystal structure of a ternary sub­complex of the MSC, DRS–AIMP2_GST_–EPRS_GST_ (the DA2E subcomplex). Unique interaction modes between DRS and AIMP2, and an ∼294 kDa decameric DA2EA3M model, were generated from our DA2E subcomplex structure. The structural information on the interactions between DRS, AIMP2 and EPRS, as well as an updated model of the MSC, provides valuable insights into the pivotal role of DRS, into the overall architecture of the MSC in higher eukaryotes and into multi-potent functions as a whole.

## 2. Materials and methods   

### 2.1. Cloning, expression and purification of the DRS–AIMP2-DX2-S34–EPRS_GST_ ternary complex   

N-terminally truncated human DRS (residues Ala21–Pro501), an N-terminally truncated human AIMP2 splice variant lacking exon 2 (residues Ser34–Gln45 and Asp115–Lys320, named AIMP2-DX2-S34) and C-terminally truncated human EPRS_GST_ (Met1–Gln157) were amplified using PCR. Each gene was cloned into pET-28a(+) vector (Novagen) to contain N-terminal, C-terminal and C-terminal His_6_ tags, respectively. The DRS, AIMP2-DX2-S34 and EPRS_GST_ cloned plasmids were separately transformed into the Rosetta2 (DE3)pLysS, BL21-CodonPlus (DE3)-RIPL and BL21-CodonPlus (DE3)-RIPL *Escherichia coli* strains, respectively. The transformed cells were grown for 16 h in Luria broth medium without induction for use as seeds in scaled-up mixture cultures. The three uninduced *E. coli* seeds containing the DRS, AIMP2-DX2-S34 or EPRS_GST_ plasmids were pooled into scaled-up culture medium to give a total seed ratio of 1:50. The scaled-up cultured cells were grown in Luria broth medium and induced by 0.5 m*M* isopropyl β-d-1-thiogalactopyranoside at an OD_600 nm_ of 0.5, followed by further incubation at 20°C for 16 h. Harvested cells were lysed by a cell sonicator (Sonics) in lysis buffer consisting of 20 m*M* Tris–HCl pH 7.5, 500 m*M* NaCl, 35 m*M* imidazole, 1 m*M* phenylmethylsulfonyl fluoride. Cell debris was removed by centrifugation at 35 000*g* for 50 min at 4°C and the supernatant was then filtered with a 0.22 µm filter to remove cell debris and any aggregated proteins. The filtered supernatant was applied onto a HiTrap Chelating HP column (GE Healthcare) equilibrated with lysis buffer for affinity chromatography. Unbound or weakly bound proteins were removed from the HiTrap Chelating HP column by six column-volume elutions with washing buffer consisting of 20 m*M* Tris–HCl pH 7.5, 500 m*M* NaCl, 50 m*M* imidazole. The retained proteins were eluted by gradually increasing addition of a buffer consisting of 20 m*M* Tris–HCl pH 7.5, 500 m*M* NaCl, 500 m*M* imidazole. The eluted protein samples were further purified by size-exclusion chromatography on a HiLoad 16/600 Superdex 200 pg column (GE Healthcare) equilibrated with a buffer consisting of 50 m*M* HEPES–NaOH pH 8.0, 200 m*M* NaCl, 5 m*M* DTT, 1%(*v*/*v*) glycerol. The purified DRS–AIMP2-DX2-S34–EPRS_GST_ complex was concentrated to 6.0 mg ml^−1^ for crystallization.

Phospho-ablative (AIMP2_GST_ S156A) and phosphor-mimetic (AIMP2_GST_ S156D and S156E) mutations were introduced into AIMP2-DX2-S34 by site-directed mutagenesis using the QuikChange II Site-Directed Mutagenesis Kit (Agilent Technologies) and the proteins were purified as described above.

Metal analysis of the purified DRS–AIMP2-DX2-S34–EPRS_GST_ complex was carried out by inductively coupled plasma mass spectrometry (ICP-MS; 820MS, Analytik Jena).

### 2.2. Crystallization and structure determination   

Initial crystals of the DA2E subcomplex were grown at 22°C by the sitting-drop vapor-diffusion method by mixing equal volumes of the protein at 6.0 mg ml^−1^ and a crystallization solution consisting of 1.2 *M* sodium phosphate monobasic, 0.8 *M* potassium phosphate dibasic, 0.1 *M* CAPS pH 10.5, 0.2 *M* lithium sulfate. DA2E crystals were optimized for X-ray data collection at 22°C using the hanging-drop vapor-diffusion method by mixing equal volumes of protein solution and crystallization solution consisting of 1.3 *M* sodium phosphate monobasic, 0.5 *M* potassium phosphate dibasic, 0.1 *M* CAPS pH 10.5, 0.5 *M* lithium sulfate with initial crystals as a seed stock and adding 0.015 m*M* CYMAL-7.

The crystals grown in the sitting drop were already cryoprotected by the reservoir solution and were flash-cooled in a nitrogen-gas stream at 100 K. Data were collected from the crystals to 3.6 Å resolution. Raw X-ray diffraction data were indexed and scaled using the *HKL*-2000 suite (Otwinowski & Minor, 1997 ▸). The crystal structure of DRS–AIMP2-DX2-S34–EPRS_GST_ was determined by molecular replacement with *MOLREP* (Vagin & Teplyakov, 2010 ▸) using the refined structures of DRS, AIMP2 and EPRS_GST_ as phasing models.

Model building of the crystal structures was carried out with *Coot* (Emsley *et al.*, 2010 ▸) and further refinement was implemented in *LORESTR* (Kovalevskiy *et al.*, 2016 ▸) and *REFMAC*5 (Murshudov *et al.*, 2011 ▸) in the *CCP*4 suite (Winn *et al.*, 2011 ▸) and in *phenix.refine* (Afonine *et al.*, 2012 ▸). Extra electron densities found around the modeled DA2E complex were carefully analyzed before manually assigning Zn^2+^ ions, phosphate ions or water molecules. Zn^2+^ ions and most of the phosphate ions were modeled based on our ICP-MS results and a binding model of DRS–tRNA^Asp^ predicted from the structure of the yeast DRS–tRNA^Asp^ complex (PDB entry 1asy; Ruff *et al.*, 1991 ▸), respectively. For modeling of water molecules, a few representative programs, such as *Coot*, *REFMAC*5 and *phenix.refine*, were implemented but could not assign water molecules that were commonly confirmed by the programs. Thus, only 12 water molecules were manually assigned by iterative placement and confirmation with subsequent refinement. Validation of the crystal structures was implemented in *MolProbity* (Chen *et al.*, 2010 ▸) and the PDB Validation Server. Data-collection and refinement statistics are summarized in Table 1 ▸. The coordinates and structure factors for the DA2E subcomplex have been deposited in the Protein Data Bank (http://www.rcsb.org) with code 6iy6.

## 3. Results   

### 3.1. *In vitro* MSC subcomplex formation: attempts to obtain a stable composition   

In order to elucidate the overall architecture of the MSC, we attempted the purification and crystallization of various MSC subcomplexes with respect to DRS, the structure of which had already been determined (Kim *et al.*, 2013 ▸). Among the MSC components, the C-terminal domain of AIMP2 is known to bind DRS (Quevillon *et al.*, 1999 ▸), but the actual interaction mode between AIMP2 and DRS remained unclear. We observed that DRS forms binary, tertiary, quaternary and pentamery subcomplexes with the GST domains of AIMP2, EPRS, MRS and AIMP3. Importantly, an AIMP2 splicing variant lacking exon 2 (AIMP2-DX2) was included in our trial because AIMP2-DX2 contains the C-terminal GST domain. To obtain sufficiently pure extracts of AIMP2-DX2 for complex structure determination, an N-terminal thioredoxin (Trx) tag was incorporated. When purified Trx-AIMP2-DX2 was subjected to Trx-tag cleavage by incubation with thrombin, the cleaved product seemed to have a smaller molecular weight (∼24 kDa) than the anticipated AIMP2-DX2 construct (∼28 kDa) as analyzed by SDS–PAGE (data not shown). Further analysis by mass spectrometry revealed that thrombin treatment removed a segment of the N-terminal peptide of the AIMP2-DX2 protein along with the Trx tag. We confirmed that the first five N-terminal residues of the cleaved AIMP2-DX2 were Ser-Tyr-Gly-Pro-Ala by N-terminal sequencing, which corresponds to residues 34–38 of AIMP2. We named the 33-amino-acid-truncated form of AIMP2 AIMP2-DX2-S34. This particular truncated form of AIMP2 was likewise observed in the mass-spectrometric analysis of MSC extracted from a human cell culture (Park *et al.*, 2015 ▸), thus justifying our trial of AIMP2-DX2-S34 incorporation into MSC subcomplexes.

**Figure 1:**
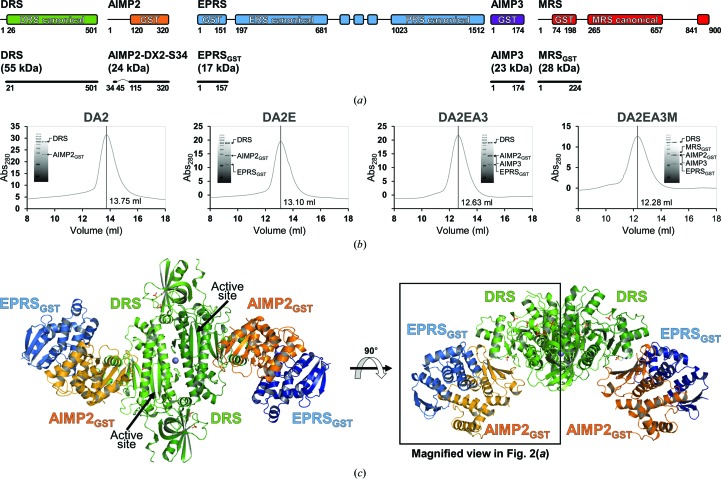
Structure and assembly of the DRS–AIMP2_GST_–EPRS_GST_ subcomplex of the MSC. (*a*) Domain compositions of DRS, AIMP2, EPRS, AIMP3 and MRS (top), and representations of the constructs used in this study (bottom). (*b*) Size-exclusion chromatography analyses of the MSC subcomplexes DA2, DA2E, DA2EA3 and DA2EA3M. Inset: SDS–PAGE analysis of the highest peak. (*c*) Left: overall view of the crystal structure of the DA2E subcomplex of the MSC in cartoon representation. DRS (green), AIMP2_GST_ (orange), EPRS_GST_ (blue) and a Zn atom (gray) are shown. Right: the DA2E structure is horizontally rotated by 90°, showing the interfaces among the components. Phosphate ions are represented as stick models. (See also Supplementary Fig. S1.)

Because the AIMP2-DX2-S34 construct was more stable in solution than its full-length counterpart AIMP2-DX2, it was used for MSC subcomplex formation along with DRS, EPRS_GST_, AIMP3 and MRS_GST_, each with either N-terminal or C-terminal His_6_ tags [Fig. 1 ▸(*a*)]. The plasmids carrying each of the proteins were cloned into separate expression strains and the expression strains were then collected together for a scaled-up culture. The proteins were only allowed to form complexes upon cell disruption by sonication. Nevertheless, the proteins stably formed the following MSC subcomplexes as verified by size-exclusion chromatography and SDS–PAGE analyses [Fig. 1 ▸(*b*)]: DRS–AIMP2-DX-S34 (DA2), DRS–AIMP2-DX2-S34–EPRS_GST_ (DA2E), DRS–AIMP2-DX2-S34–EPRS_GST_–AIMP3 (DA2EA3) and DRS–AIMP2-DX2-S34–EPRS_GST_–AIMP3–MRS_GST_ (DA2EA3M). Of the four MSC subcomplexes that were purified, the DA2E sub­complex (∼192 kDa) was successfully crystallized for structure determination.

### 3.2. The DRS–AIMP2_GST_–EPRS_GST_ (DA2E) subcomplex is orientated around the DRS homodimer   

We successfully determined the tertiary complex structure of DA2E at 3.6 Å resolution by the molecular-replacement (MR) method using the structures of DRS and the AIMP2_GST_–EPRS_GST_ binary complex (PDB entries 4j15 and 5a34; Cho *et al.*, 2015 ▸; Kim *et al.*, 2013 ▸) as phasing models. Despite our efforts to elucidate the structure of AIMP2 with the exon 1 region, only the GST domain of AIMP2 corresponding to exons 3 and 4 (residues 117–320) could be determined (Supplementary Fig. S2). Therefore, in the following we will use the term AIMP2_GST_ instead of AIMP2-DX2-S34 to refer to the protein. The DA2E crystals belonged to space group *P*6_1_ and contained two DA2E subcomplexes in the asymmetric unit, each comprising two molecules of DRS, AIMP2_GST_ and EPRS_GST_.

The DA2E components are oriented around the central DRS homodimer in a twofold rotational symmetry with the center of mass of DA2E located in the DRS dimeric interface [Fig. 1 ▸(*c*)]. The DRS structure in the DA2E complex did not exhibit noticeable structural changes compared with that of DRS alone, in that the anticodon-binding domain (residues 57–146) and the catalytic domain (residues 189–497) of DRS could be well modeled. However, the hinge region between the anticodon-binding domain and the catalytic domain (residues 155–178), as well as two other loop regions (residues 225–248 and 273–281) that are known to bind tRNA^Asp^ in an induced-fit manner (Sauter *et al.*, 2000 ▸), could not be modeled owing to a lack of electron density [Supplementary Fig. S1(*a*)]. Extra electron density around the DA2E complex could not be clearly discerned owing to the low resolution limit and were manually assigned with discretion considering their environment. Extra spherical electron densities observed at the DRS dimeric interfaces were coordinated by two His204/Glu208 pairs, each from one monomer, and were modeled as Zn^2+^ ions according to the metal-content analysis results using ICP-MS (Supplementary Fig. S3). Extra electron densities observed at the tRNA^Asp^-binding sites of DRS molecules (catalytic sites and anticodon-binding sites) were modeled as phosphate ions present in the crystallization solution at high concentrations, which seem to mimic the phosphate moieties of the tRNA^Asp^ backbone (Supplementary Fig. S4). Extra electron densities observed at the DRS–AIMP2_GST_ interfaces were also modeled as phosphate ions which interact with backbone O and N atoms of the nearby DRS. Since the interactions do not seem to be biologically significant and the phosphate ions were only introduced for crystallization after *in vitro* DA2E complex formation, the phosphate ions might have contributed to further stabilizing the DA2E complexes in the crystal. Modeling water molecules using modeling programs did not produce consistent results owing to the low resolution limit. After a careful iterative process, only a dozen water molecules were modeled.

AIMP2_GST_ and EPRS_GST_ both adopt a GST fold consisting of an N-terminal Trx fold and a C-terminal α-helical sub­domain. EPRS_GST_ adopts a canonical four-stranded β-sheet in its Trx subdomain, whereas AIMP2_GST_ has an extra stretch of peptide (residues 148–170) which constitutes a strand–loop–helix motif (β2–α2) following helix α1, thus comprising a characteristic five-stranded β-sheet [Supplementary Fig. S1(*b*)]. Residues 171–179 and 289–291 of AIMP2_GST_ in the α2–β3 loop and the α6–α7 loop, respectively, could not be modeled owing to a lack of electron density [Supplementary Fig. S1(*b*)].

**Figure 2:**
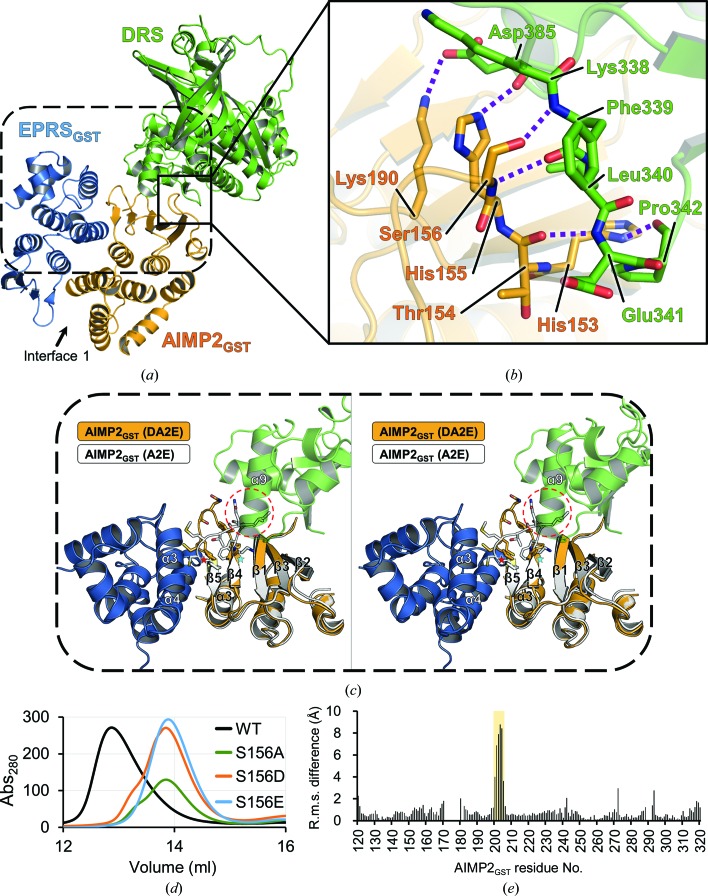
Interfaces among the components of the DA2E subcomplex. (*a*) Overall view of the DA2E structure showing the interfaces between DRS and AIMP2_GST_ and between AIMP2_GST_ and EPRS_GST_. (*b*) Close-up view of interactions between DRS (green) and AIMP2_GST_ (orange). Hydrogen bonds and salt bridges between DRS and AIMP2_GST_ are indicated by purple dashed lines. (*c*) Stereoscopic representation showing the effect of DRS incorporation on the AIMP2_GST_–EPRS_GST_ interface. The AIMP2_GST_ structure from A2E (PDB entry 5a34; white) was superposed onto the DA2E structure (orange) to show conformational change in the β4–β5 loop. The β4–β5 loop region of AIMP2_GST_ from the A2E subcomplex (red circle) collides with helix α9 of DRS shown in transparent cartoon representation (green). The first and the last residues of the β4–β5 loop (Lys198 and Pro206) are marked with cyan and red stars, respectively. (*d*) Size-exclusion chromatography analyses of Ser156 mutants on DA2 complex formation. (*e*) R.m.s.d. analysis of AIMP2_GST_ structures in DA2E and the heterodimeric complex, showing remarkably large differences in the structures at the β4–β5 loop (Lys198–Pro206), highlighted in a yellow box.

*Protein Interfaces, Surfaces and Assemblies* (*PISA*) analysis (Krissinel & Henrick, 2007 ▸) of the interfaces in the DA2E subcomplex structure revealed that interactions between DRS and AIMP2_GST_ arise mainly from residues in the α7–β9 loop (residues 338–342) and the C-terminus of helix α9 (residues 383 and 384) of DRS and in the N-terminal subdomain of AIMP2_GST_, which comprises a β-sheet [Figs. 2 ▸(*a*) and 2 ▸(*b*)]. The interaction between AIMP2_GST_ and EPRS_GST_ is mainly through the heterodimerization of GST domains, in which the proteins are related to each other by a twofold rotational symmetry. In the following sections, the interfaces between the components of the DA2E subcomplex will be extensively described.

### 3.3. Ser156 of AIMP2 plays a key role in the interaction between DRS and AIMP2_GST_ via hydrogen bonds   

The DA2E structure revealed that the binding interfaces of DRS and AIMP2_GST_ have an average area of 747.2 Å^2^ as calculated by *PISA*. The AIMP2-binding motif of DRS is located in the middle of the DRS catalytic domain, discrete from the DRS dimeric interface, the tRNA^Asp^-binding site and the active site [Fig. 1 ▸(*c*)], which is in accordance with the fact that ARSs retain aminoacyl-tRNA synthetase activities in the MSC (Mirande *et al.*, 1985 ▸).

The complex formation of DRS and AIMP2_GST_ is mainly mediated by two binding interfaces [Fig. 2 ▸(*a*)]. The major interface is formed by multiple hydrogen bonds between Lys338–Pro342 in the α7–β9 loop of DRS and His153–Ser156 in the β2–α2 loop of AIMP2_GST_ [Fig. 2 ▸(*b*)], while the other interface involves helix α9 of DRS and the β-sheet of AIMP2_GST_ [Fig. 2 ▸(*c*)]. The loops in the major interface establish multiple hydrogen bonds between the C^α^ backbones, which are reminiscent of the hydrogen-bonding networks of an antiparallel β-sheet. Interestingly, Ser156 of AIMP2_GST_ seems to play a crucial role in mediating the interaction between DRS and AIMP2_GST._ The side chain of Ser156 acts as a hydrogen-bond donor coupled with the backbone amide group of Phe339 of DRS, while the backbone amide group of Ser156 forms a hydrogen bond to the backbone O atom of Phe339 of DRS [Fig. 2 ▸(*b*)]. Ser156 has previously been shown to be exposed to the solvent and phosphorylated by TGF-β signaling. Phosphorylation of Ser156 induced dissociation of AIMP2 from the MSC, followed by translocation into the cell nucleus, where AIMP2 functions as a tumor suppressor in concert with Smurf2 (Kim *et al.*, 2016 ▸).

Two mutants, AIMP2_GST_ S156D and S156E, were generated to mimic the phosphorylation of Ser156, and the AIMP2_GST_ S156A mutant was generated to abolish the side-chain-mediated hydrogen bond. The interaction between the AIMP2_GST_ mutants and DRS was evaluated by size-exclusion chromatography [Fig. 2 ▸(*d*)]. All three AIMP2_GST_ mutants could not form binary complexes with DRS, which suggests that the hydrogen bond from the side chain of AIMP2 Ser156 is crucial for the interaction with DRS and that Ser156 phosphorylation would disintegrate the MSC assembly.

### 3.4. The DA2E ternary complex accompanies a conformational change in interface 1 between two GST domains   

*PISA* analysis of the DA2E structure revealed that the binding interfaces of AIMP2_GST_ and EPRS_GST_ have an average area of 1083.3 Å^2^, which is larger than that between DRS and AIMP2_GST_. The networks of interactions between heterodimeric GST domains in the MSC have been extensively analyzed previously, and were assigned as Interface 1 and Interface 2 (Cho *et al.*, 2015 ▸). Here, we briefly describe the nature of Interface 1 between AIMP2_GST_ and EPRS_GST_ and intensively analyze a unique conformational change therein elicited by the incorporation of DRS compared with the heterodimeric A2E structure.

Interface 1 is formed by the bundling of helices α3 and α4 of AIMP2_GST_ and α2 and α3 of EPRS_GST_, which establish multiple intermolecular hydrogen bonds and salt bridges. When the structures of AIMP2_GST_ in the DA2E subcomplex and in the heterodimeric A2E subcomplex (PDB entry 5a34; Cho *et al.*, 2015 ▸) were compared, the two structures were very similar over 201 equivalent C^α^ atoms, with a root-mean-square deviation (r.m.s.d.) of 0.87 Å. Although the constructs used for this comparison were truncated differently, the GST-domain structure was not affected by the truncations. However, a remarkably high r.m.s.d. was observed for residues Phe199–Pro206, with a maximum C^α^ deviation of 8.78 Å at Thr203 [Fig. 2 ▸(*e*)]. These residues belong to the β4–β5 loop, the last three residues of which (Met204-Cys205-Pro206) make hydrophobic contacts with helix α4 of EPRS_GST_ [Fig. 2 ▸(*c*)]. Incorporation of DRS causes a major conformational change in the β4–β5 loop, where the C-terminal end of helix α9 of DRS rests on the β-sheet of AIMP2_GST_ [Fig. 2 ▸(*c*), dashed red circle]. Superposing the AIMP2 structure from the A2E subcomplex [Fig. 2 ▸(*c*), white] (PDB entry 5a34) onto our AIMP2_GST_ structure [Fig. 2 ▸(*c*), orange] reveals that helix α9 of DRS occupies the region where the β4–β5 loop would be in the A2E subcomplex structure. The results of *PISA* analysis show that residues Glu382-Lys383-Tyr384 on helix α9 of DRS make mainly hydrophobic contacts with Leu119, Val123 and Ile201 of AIMP2_GST_, while the β4–β5 loop of AIMP2_GST_ is sandwiched between DRS and EPRS_GST_ to adopt a more ordered β-turn structure [Fig. 2 ▸(*c*), orange]. Consequent rearrangement of the C^α^ backbone in the β4–β5 loop elicited a remarkable difference in Interface 1 compared with that in the heterodimeric A2E complex.

### 3.5. Adjacent DA2E subcomplexes are bridged via Interface 2 between two EPRS_GST_ molecules   

**Figure 3:**
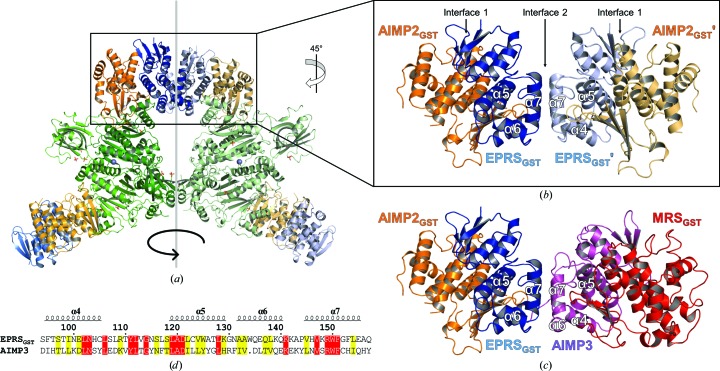
Interaction between GST domains via Interfaces 1 and 2. (*a*) Two DA2E subcomplexes within an asymmetric unit show an overall twofold rotational symmetry. One of the subcomplexes is shown in paler colors to distinguish the two. (*b*) Close-up view of the heterodimeric AIMP2_GST_–EPRS_GST_ complexes at the interface of the two DA2E subcomplexes. AIMP2_GST_ and EPRS_GST_ interact via Interface 1, while two EPRS_GST_ molecules from adjacent subcomplexes interact via Interface 2. (*c*) Close-up view of the model structure of heterotetrameric AIMP2_GST_–EPRS_GST_–AIMP3–MRS_GST_ built upon the DA2E subcomplex structure, in which AIMP3 and MRS_GST_ are colored violet and red, respectively (PDB entry 4bvx). The assembly of the heterotetrameric GST domains is identical to that of AIMP2_GST_–EPRS_GST_–EPRS_GST_–AIMP2_GST_ observed in our structure (*b*). (*d*) Sequence alignment of residues in Interface 2 of EPRS_GST_ and AIMP3 using *Clustal Omega* (Sievers *et al.*, 2011 ▸) displayed with *ESPript* 3 (Robert & Gouet, 2014 ▸). The secondary-structure elements were defined based on the structure of EPRS_GST_. Conserved residues and similar residues are highlighted in red and yellow, respectively.

To our surprise, we observed two molecules of EPRS_GST_ interacting via Interface 2, each of which are from adjacent DA2E subcomplexes in the asymmetric unit [Figs. 3 ▸(*a*) and 3 ▸(*b*)]. In the case of Interface 2 of EPRS_GST_ and AIMP3 (PDB entry 4bvx; Cho *et al.*, 2015 ▸), residues from helices α7 and the α4–α5 loops from each of the GST domains similarly establish a complex network via hydrogen bonds and salt bridges [Fig. 3 ▸(*c*)] to the EPRS_GST_ crystallographic interface. Because EPRS_GST_ and AIMP3 have a high overall sequence similarity of 40.1% [Fig. 3 ▸(*d*)] and share the same structural domain [Figs. 3 ▸(*b*) and 3 ▸(*c*)], it is understandable that the exposed surface of EPRS_GST_ would facilitate dimerization via Interface 2 in the absence of the legitimate binding partner AIMP3.

During our trial crystallization of MSC subcomplexes, we obtained crystals of the binary DA2 subcomplex, which however only diffracted to 4.5 Å resolution (data not shown), compared with crystals of the ternary DA2E subcomplex, which diffracted to 3.6 Å resolution. While DA2 subcomplex molecules were packed with no apparent noncrystallographic symmetry within the asymmetric unit, the DA2E subcomplex molecules in the asymmetric unit are related to each other by a twofold rotational symmetry [Fig. 3 ▸(*a*)]. Although the interaction between two EPRS_GST_ molecules from adjacent DA2E subcomplexes within the asymmetric unit would have been a crystallographic artifact, it is clear that this interaction contributed to the stabilization and hence successful structure determination of the DA2E subcomplex.

## 4. Discussion   

The MSC is a multi-protein complex composed of nine ARSs and three AIMPs. It has been proposed that the congregation of individual ARSs and scaffold proteins would facilitate the translation process by providing a constant flow of amino-acid-charged tRNAs (Negrutskii & Deutscher, 1991 ▸). In this work, we report a pivotal architecture of the MSC comprising two AIMP2_GST_–EPRS_GST_ heterodimers which extend from the central DRS homodimer. While searching for an optimum construct of AIMP2 for DA2E structure determination, we made the serendipitous discovery of AIMP2-DX2 lacking the first 33 residues (AIMP2-DX2-S34), the incorporation of which into the MSC was known to be biologically significant (Park *et al.*, 2015 ▸). This construct seemed to have greatly contributed to the structure determination of DA2E because the flexible N-terminal region of AIMP2 was removed, which could facilitate the crystallization of the DA2E ternary complex.

Following the structure determination of DA2E, the inter­actions among the DA2E components were intensively inspected. The interaction between DRS and AIMP2_GST_ is uniquely mediated by the β-sheet of AIMP2_GST_ and its adjoining loops, while the interaction between AIMP2_GST_ and EPRS_GST_ is mediated via the heterodimeric GST-domain Interface 1, as previously reported (Cho *et al.*, 2015 ▸). By analyzing the interface between DRS and AIMP2_GST_, we speculated that Ser156 of AIMP2_GST_ is a key residue in complex formation and demonstrated this by size-exclusion chromatography with relevant AIMP2_GST_ mutants. To our surprise, both AIMP2_GST_ phosphor-ablative (AIMP2_GST_ S156A) and phosphor-mimetic (AIMP2_GST_ S156D and S156E) mutants successfully inhibited the interaction between DRS and AIMP2_GST_ [Fig. 2 ▸(*d*)]. Phosphorylation of Ser156 is known to trigger the release of AIMP2 from the MSC and subsequent translocation into the nucleus for interaction with Smurf2 to suppress the nuclear export of Smurf2 and hence elicit tumor-suppressive TGF-β signaling (Kim *et al.*, 2016 ▸). Since AIMP2 is a key scaffold protein that interconnects most components (Robinson *et al.*, 2000 ▸), the release of AIMP2 from the MSC would cause breakdown of the integral MSC, which subsequently enables nontranslational functions of the dissociated components (Kim *et al.*, 2002 ▸). For example, KRS released from the MSC upon phosphorylation of its Ser206 residue is translocated from the cytoplasm into the nucleus to activate the downstream signaling pathway for diadenosine tetraphosphate (Ap_4_A) production (Yannay-Cohen *et al.*, 2009 ▸). Similar phenomena are observed for EPRS following interferon-γ activation (Sampath *et al.*, 2004 ▸), suggesting that the release of MSC components by post-translational modification is a measure of switching cellular metabolism from growth to damage control in response to a harsh environment (Lee *et al.*, 2004 ▸).

Although the mechanistic detail of AIMP2 release from the MSC is not clear, DRS would also be dissociated from the MSC along with AIMP2 as it is the only binding partner. The release of DRS might allow subsequent post-translational modifications such as acetylation, phosphorylation or ubiquitination (Kim *et al.*, 2013 ▸), which might in turn trigger as yet unknown nontranslational functions of DRS. Overall, post-translational modification and subsequent repositioning of the components of MSC could be a common method of eliciting nontranslational functions.

**Figure 4:**
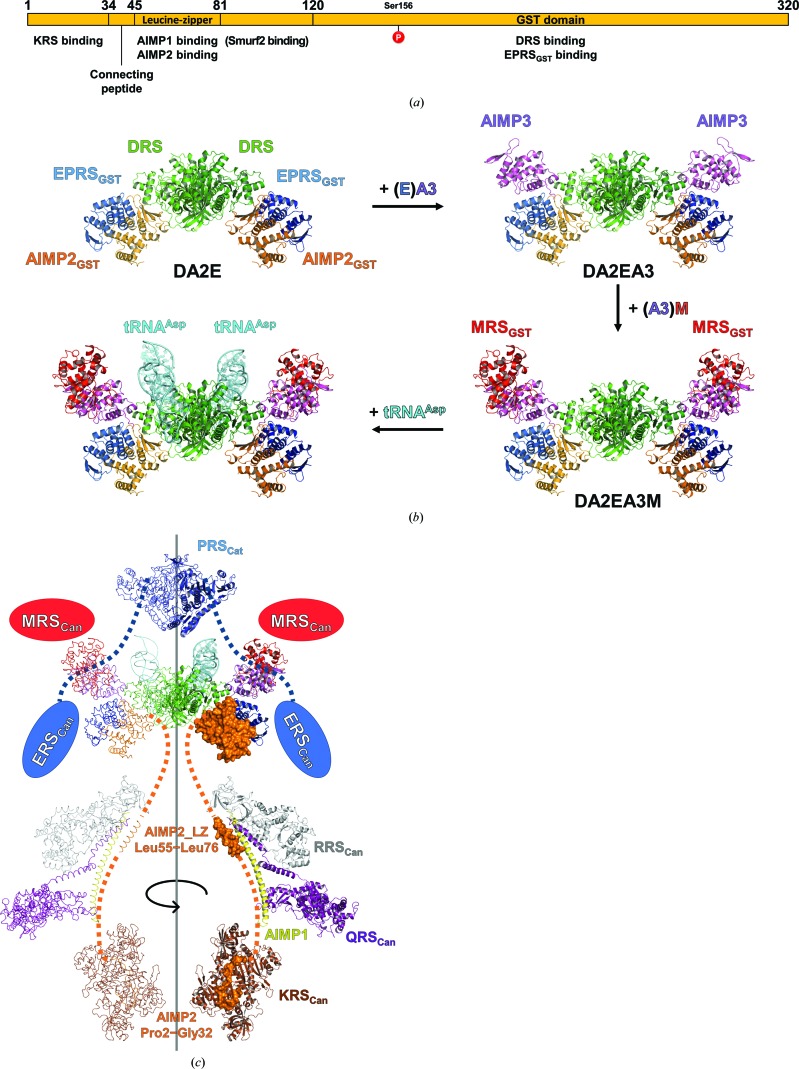
Model building of the MSC subcomplex based on the DA2E structure. (*a*) Schematic of the AIMP2 sequence showing multiple protein-binding sites as a scaffold protein. Notable structural motifs are indicated within the schematic. The names of the proteins are listed below the region of AIMP2 to which they bind. The site of phosphorylation (Ser156) is marked with a red circle. (*b*) Building the DA2EA3M structure model onto the DA2E structure. The structures of EPRS_GST_–AIMP3 (PDB entry 5bmu) and AIMP3–MRS_GST_ (PDB entry 4bvx) complexes were sequentially added to the DA2E structure by superposition. The yeast tRNA^Asp^ structure was laid onto the model by superimposing the structure of yeast DRS in complex with yeast tRNA^Asp^ (PDB entry 1asy; Ruff *et al.*, 1991 ▸). (*c*) Structure of the extended MSC model including DA2EA3M and RQA1 (PDB entry 4r3z) subcomplexes and homodimeric KRS structures (PDB entry 4dpg). Each of the components were assigned with respect to where they would bind to AIMP2, which is represented as a cartoon model. AIMP2 segments are shown in surface representation. A symmetry-related duplicate of the extended model is shown as a ribbon representation. Flexible regions of AIMP2 and EPRS are represented by orange and blue dashed lines, respectively. The coloring scheme is as follows: DRS, green; yeast tRNA^Asp^, cyan; AIMP2, orange; EPRS, blue; AIMP3, violet; MRS, red; RRS, gray; QRS, purple; AIMP1, yellow; KRS, brown.

Previous interactome studies have shown that AIMP2 acts as a scaffold in the assembly of the MSC to harbor binding sites for other components (Quevillon *et al.*, 1999 ▸; Robinson *et al.*, 2000 ▸). KRS (Ofir-Birin *et al.*, 2013 ▸), AIMP1 (Ahn *et al.*, 2003 ▸), DRS and EPRS_GST_ described here are all known binding partners of AIMP2 [Fig. 4 ▸(*a*)]. Using previously reported information about the interactions among the MSC components, we built a model of the MSC subcomplex based on our DA2E structure. Firstly, heterotetrameric GST domains could be reasonably appended to the DA2E structure by sequentially superimposing structures of EA3 (EPRS_GST_–AIMP3; PDB entry 5bmu) and A3M (AIMP3–MRS_GST_; PDB entry 4bvy) (Cho *et al.*, 2015 ▸), resulting in the DA2EA3M pentameric subcomplex model [Fig. 4 ▸(*b*)]. The conformation of the DA2EA3M subcomplex model did not hinder the binding of tRNA^Asp^, the binding site of which was speculated from the yeast DRS–tRNA^Asp^ complex (Ruff *et al.*, 1991 ▸). For a more comprehensive model of DA2EA3M, canonical domains of ERS, PRS and MRS (ERS_Can_, PRS_Can_ and MRS_Can_, respectively) should be placed.

Although the structures of the canonical domains are known (Son *et al.*, 2013 ▸), no structural information is available for the linkers between the canonical domains and their N-terminally appending GST domains. However, considering the short lengths of the peptides connecting EPRS_GST_ to ERS_Can_ and MRS_GST_ to MRS_Can_ (approximately 40 amino acids each), we could at least surmise that these canonical domains would reside next to their respective GST domains, as denoted by the blue and red ovals, respectively, in Fig. 4 ▸(*c*). Assigning the position of the PRS_Can_ homodimer is more precarious. There is no structural information about the 300-amino-acid linker region between ERS_Can_ and PRS_Can_, except for that on the three tandem WHEP domains elucidated by nuclear magnetic resonance (NMR) spectroscopy (Jeong *et al.*, 2000 ▸). For our model of the DA2EA3M structure, this linker region is illustrated with dashed lines to reflect its high flexibility. The homodimeric PRS_Can_ was positioned above the DRS homodimer, as shown in Fig. 4 ▸(*c*), in such a way that a theoretical twofold rotational symmetry axis of the DRS homodimer coincides with that of the PRS_Can_ homodimer.

In contrast, AIMP1 tethers a stable trimeric arginyl-tRNA (RQA1) subcomplex (Fu *et al.*, 2014 ▸) to DA2EA3M via a coiled-coil interaction with the leucine-zipper motif of AIMP2 (residues 55–76; Quevillon *et al.*, 1999 ▸), as shown in Fig. 4 ▸(*c*). Two KRS homodimers were additionally included in the MSC subcomplex model by estimating the relative position of the N-terminal region of AIMP2 (Gly2–Pro33), which was present in the structure of the KRS homodimer, to the leucine-zipper motif.

However, assembling the components of the MSC onto an AIMP2 molecule according to the above interaction data would only result in one half of the whole MSC complex because most of the components of the MSC, including AIMP2 itself, exist as duplicates (Dias *et al.*, 2013 ▸). Thus far, how two AIMP2-centered subcomplexes are brought together to compose the complete MSC has remained elusive. Our DA2E subcomplex structure with a central DRS homodimer provides evidence that DRS plays a pivotal role in the assembly of the MSC by conjoining two AIMP2 molecules as well as additional components. Taken together, our DA2E subcomplex structure provides valuable new structural information about the DRS-centered DA2E subcomplex and further extends our knowledge on the overall assembly of the MSC to thoroughly elucidate the structure, dynamics and synergistic effects of the MSC.

## Supplementary Material

PDB reference: human cytosolic aspartyl-tRNA synthetase in complex with glutathione *S*-transferase domains from aminoacyl-tRNA synthase complex-interacting multifunctional protein 2 and glutamyl-prolyl-tRNA synthetase, 6iy6

Supplementary Figures. DOI: 10.1107/S2052252519010790/lz5028sup1.pdf

## Figures and Tables

**Table 1:** Data-collection and refinement statistics Values in parentheses are for the highest resolution shell.

PDB code	6iy6
Diffraction source	PLS-5C
Wavelength (Å)	0.9790
Temperature (K)	100
Space group	*P*6_1_
*a*, *b*, *c* (Å)	108.07, 108.07, 815.64
α, β, γ (°)	90, 90, 120
Resolution range (Å)	50.000–3.600 (3.660–3.600)
Total No. of reflections	177735
No. of unique reflections	61327
Completeness (%)	98.7 (99.3)
Multiplicity	2.9 (2.9)
〈*I*/σ(*I*)〉	8.182 (1.038)†
*R*_meas_	0.126 (0.949)
*R*_p.i.m._	0.072 (0.544)
Overall *B* factor from Wilson plot (Å^2^)	113.5
Resolution range (Å)	49.5100–3.6000 (3.6910–3.5980)
Completeness (%)	98.8
No. of reflections, working set	58167 (4234)
No. of reflections, test set	3084 (250)
Final *R*_work_	0.237 (0.332)
Final *R*_free_	0.275 (0.346)
No. of non-H atoms	
Protein	24440
Phosphate	85
Zn^2+^	2
Water	12
R.m.s. deviations	
Bonds (Å)	0.002
Angles (°)	1.179
Average *B* factors (Å^2^)	
Protein	144.2
Phosphate	175.7
Zn^2+^	140.6
Water	65.1
Ramachandran plot	
Most favored (%)	94.41
Allowed (%)	5.52

† 〈*I*/σ(*I*)〉 in the resolution range 3.88–3.60 Å falls below 2.0 but remains above 1.0. Reflections in this resolution range could be justified by reasonable linear *R*-factor (0.512–0.772) and CC_1/2_ (0.756–0.541) values.

## References

[bb1] Afonine, P. V., Grosse-Kunstleve, R. W., Echols, N., Headd, J. J., Moriarty, N. W., Mustyakimov, M., Terwilliger, T. C., Urzhumtsev, A., Zwart, P. H. & Adams, P. D. (2012). *Acta Cryst.* D**68**, 352–367. 10.1107/S0907444912001308 PMC3322595 22505256

[bb2] Ahn, H.-C., Kim, S. & Lee, B.-J. (2003). *FEBS Lett.* **542**, 119–124. 10.1016/s0014-5793(03)00362-4 12729910

[bb3] Berg, P. & Offengand, E. J. (1958). *Proc. Natl Acad. Sci. USA*, **44**, 78–86. 10.1073/pnas.44.2.78 PMC335368 16590176

[bb4] Chen, V. B., Arendall, W. B., Headd, J. J., Keedy, D. A., Immormino, R. M., Kapral, G. J., Murray, L. W., Richardson, J. S. & Richardson, D. C. (2010). *Acta Cryst.* D**66**, 12–21. 10.1107/S0907444909042073 PMC2803126 20057044

[bb5] Cho, H. Y., Maeng, S. J., Cho, H. J., Choi, Y. S., Chung, J. M., Lee, S., Kim, H. K., Kim, J. H., Eom, C.-Y., Kim, Y.-G., Guo, M., Jung, H. S., Kang, B. S. & Kim, S. (2015). *J. Biol. Chem.* **290**, 29313–29328. 10.1074/jbc.M115.690867 PMC4705937 26472928

[bb6] Choi, J. W., Kim, D. G., Lee, A.-E., Kim, H. R., Lee, J. Y., Kwon, N. H., Shin, Y. K., Hwang, S.-K., Chang, S.-H., Cho, M.-H., Choi, Y.-L., Kim, J., Oh, S. H., Kim, B., Kim, S.-Y., Jeon, H.-S., Park, J. Y., Kang, H. P., Park, B. J., Han, J. M. & Kim, S. (2011). *PLoS Genet.* **7**, e1001351. 10.1371/journal.pgen.1001351 PMC3069106 21483803

[bb7] Dias, J., Renault, L., Pérez, J. & Mirande, M. (2013). *J. Biol. Chem.* **288**, 23979–23989. 10.1074/jbc.M113.489922 PMC3745343 23836901

[bb8] Emsley, P., Lohkamp, B., Scott, W. G. & Cowtan, K. (2010). *Acta Cryst.* D**66**, 486–501. 10.1107/S0907444910007493 PMC2852313 20383002

[bb9] Fu, Y., Kim, Y., Jin, K. S., Kim, H. S., Kim, J. H., Wang, D., Park, M., Jo, C. H., Kwon, N. H., Kim, D., Kim, M. H., Jeon, Y. H., Hwang, K. Y., Kim, S. & Cho, Y. (2014). *Proc. Natl Acad. Sci. USA*, **111**, 15084–15089. 10.1073/pnas.1408836111 PMC4210331 25288775

[bb10] Guo, M., Yang, X.-L. & Schimmel, P. (2010). *Nat. Rev. Mol. Cell Biol.* **11**, 668–674. 10.1038/nrm2956 PMC3042954 20700144

[bb11] Han, J. M., Jeong, S. J., Park, M. C., Kim, G., Kwon, N. H., Kim, H. K., Ha, S. H., Ryu, S. H. & Kim, S. (2012). *Cell*, **149**, 410–424. 10.1016/j.cell.2012.02.044 22424946

[bb12] Han, J. M., Lee, M. J., Park, S. G., Lee, S. H., Razin, E., Choi, E.-C. & Kim, S. (2006). *J. Biol. Chem.* **281**, 38663–38667. 10.1074/jbc.M605211200 17062567

[bb13] Han, J. M., Park, B.-J., Park, S. G., Oh, Y. S., Choi, S. J., Lee, S. W., Hwang, S.-K., Chang, S.-H., Cho, M.-H. & Kim, S. (2008). *Proc. Natl Acad. Sci. USA*, **105**, 11206–11211. 10.1073/pnas.0800297105 PMC2516205 18695251

[bb14] Jeong, E.-J., Hwang, G.-S., Kim, K. H., Kim, M. J., Kim, S. & Kim, K.-S. (2000). *Biochemistry*, **39**, 15775–15782. 10.1021/bi001393h 11123902

[bb15] Kim, D. G., Lee, J. Y., Lee, J.-H., Cho, H. Y., Kang, B. S., Jang, S.-Y., Kim, M. H., Guo, M., Han, J. M., Kim, S.-J. & Kim, S. (2016). *Cancer Res.* **76**, 3422–3436. 10.1158/0008-5472.CAN-15-3255 27197155

[bb16] Kim, J. Y., Kang, Y.-S., Lee, J.-W., Kim, H. J., Ahn, Y. H., Park, H., Ko, Y.-G. & Kim, S. (2002). *Proc. Natl Acad. Sci. USA*, **99**, 7912–7916.

[bb17] Kim, K. R., Park, S. H., Kim, H. S., Rhee, K. H., Kim, B.-G., Kim, D. G., Park, M. S., Kim, H.-J., Kim, S. & Han, B. W. (2013). *Proteins*, **81**, 1840–1846. 10.1002/prot.24306 PMC3824080 23609930

[bb18] Ko, Y.-G., Kang, Y.-S., Kim, E.-K., Park, S. G. & Kim, S. (2000). *J. Cell Biol.* **149**, 567–574. 10.1083/jcb.149.3.567 PMC2174846 10791971

[bb19] Ko, Y.-G., Kim, E.-Y., Kim, T., Park, H., Park, H.-S., Choi, E.-J. & Kim, S. (2001). *J. Biol. Chem.* **276**, 6030–6036. 10.1074/jbc.M006189200 11096076

[bb20] Kovalevskiy, O., Nicholls, R. A. & Murshudov, G. N. (2016). *Acta Cryst.* D**72**, 1149–1161. 10.1107/S2059798316014534 PMC5053141 27710936

[bb21] Krissinel, E. & Henrick, K. (2007). *J. Mol. Biol.* **372**, 774–797. 10.1016/j.jmb.2007.05.022 17681537

[bb22] Kyriacou, S. V. & Deutscher, M. P. (2008). *Mol. Cell*, **29**, 419–427. 10.1016/j.molcel.2007.11.038 PMC2273998 18313381

[bb23] Lee, S. W., Cho, B. H., Park, S. G. & Kim, S. (2004). *J. Cell Sci.* **117**, 3725–3734. 10.1242/jcs.01342 15286174

[bb24] Lo, W.-S., Gardiner, E., Xu, Z., Lau, C.-F., Wang, F., Zhou, J. J., Mendlein, J. D., Nangle, L. A., Chiang, K. P., Yang, X.-L., Au, K.-F., Wong, W. H., Guo, M., Zhang, M. & Schimmel, P. (2014). *Science*, **345**, 328–332. 10.1126/science.1252943 PMC4188629 25035493

[bb25] Mirande, M., Le Corre, D. & Waller, J. P. (1985). *Eur. J. Biochem.* **147**, 281–289. 10.1111/j.1432-1033.1985.tb08748.x 3971983

[bb26] Murshudov, G. N., Skubák, P., Lebedev, A. A., Pannu, N. S., Steiner, R. A., Nicholls, R. A., Winn, M. D., Long, F. & Vagin, A. A. (2011). *Acta Cryst.* D**67**, 355–367. 10.1107/S0907444911001314 PMC3069751 21460454

[bb27] Negrutskii, B. S. & Deutscher, M. P. (1991). *Proc. Natl Acad. Sci. USA*, **88**, 4991–4995. 10.1073/pnas.88.11.4991 PMC51793 2052582

[bb28] Ofir-Birin, Y., Fang, P., Bennett, S. P., Zhang, H.-M., Wang, J., Rachmin, I., Shapiro, R., Song, J., Dagan, A., Pozo, J., Kim, S., Marshall, A. G., Schimmel, P., Yang, X.-L., Nechushtan, H., Razin, E. & Guo, M. (2013). *Mol. Cell*, **49**, 30–42. 10.1016/j.molcel.2012.10.010 PMC3766370 23159739

[bb29] Otwinowski, Z. & Minor, W. (1997). *Methods Enzymol.* **276**, 307–326. 10.1016/S0076-6879(97)76066-X 27754618

[bb30] Park, S.-J., Ahn, H. S., Kim, J. S. & Lee, C. (2015). *PLoS One*, **10**, e0142253. 10.1371/journal.pone.0142253 PMC4636271 26544075

[bb31] Quevillon, S., Robinson, J. C., Berthonneau, E., Siatecka, M. & Mirande, M. (1999). *J. Mol. Biol.* **285**, 183–195. 10.1006/jmbi.1998.2316 9878398

[bb32] Rho, S. B., Kim, M. J., Lee, J. S., Seol, W., Motegi, H., Kim, S. & Shiba, K. (1999). *Proc. Natl Acad. Sci. USA*, **96**, 4488–4493. 10.1073/pnas.96.8.4488 PMC16359 10200289

[bb33] Robert, X. & Gouet, P. (2014). *Nucleic Acids Res.* **42**, W320–W324. 10.1093/nar/gku316 PMC4086106 24753421

[bb34] Robinson, J. C., Kerjan, P. & Mirande, M. (2000). *J. Mol. Biol.* **304**, 983–994. 10.1006/jmbi.2000.4242 11124041

[bb35] Ruff, M., Krishnaswamy, S., Boeglin, M., Poterszman, A., Mitschler, A., Podjarny, A., Rees, B., Thierry, J. C. & Moras, D. (1991). *Science*, **252**, 1682–1689. 10.1126/science.2047877 2047877

[bb36] Sampath, P., Mazumder, B., Seshadri, V., Gerber, C. A., Chavatte, L., Kinter, M., Ting, S. M., Dignam, J. D., Kim, S., Driscoll, D. M. & Fox, P. L. (2004). *Cell*, **119**, 195–208. 10.1016/j.cell.2004.09.030 15479637

[bb37] Sauter, C., Lorber, B., Cavarelli, J., Moras, D. & Giegé, R. (2000). *J. Mol. Biol.* **299**, 1313–1324. 10.1006/jmbi.2000.3791 10873455

[bb38] Sievers, F., Wilm, A., Dineen, D., Gibson, T. J., Karplus, K., Li, W., Lopez, R., McWilliam, H., Remmert, M., Söding, J., Thompson, J. D. & Higgins, D. G. (2011). *Mol. Syst. Biol.* **7**, 539. 10.1038/msb.2011.75 PMC3261699 21988835

[bb39] Son, J., Lee, E. H., Park, M., Kim, J. H., Kim, J., Kim, S., Jeon, Y. H. & Hwang, K. Y. (2013). *Acta Cryst.* D**69**, 2136–2145. 10.1107/S0907444913020556 24100331

[bb40] Vagin, A. & Teplyakov, A. (2010). *Acta Cryst.* D**66**, 22–25. 10.1107/S0907444909042589 20057045

[bb50] Winn, M. D., Ballard, C. C., Cowtan, K. D., Dodson, E. J., Emsley, P., Evans, P. R., Keegan, R. M., Krissinel, E. B., Leslie, A. G. W., McCoy, A., McNicholas, S. J., Murshudov, G. N., Pannu, N. S., Potterton, E. A., Powell, H. R., Read, R. J., Vagin, A. & Wilson, K. S. (2011). *Acta Cryst.* D**67**, 235–242. 10.1107/S0907444910045749 PMC3069738 21460441

[bb41] Yannay-Cohen, N., Carmi-Levy, I., Kay, G., Yang, C. M., Han, J. M., Kemeny, D. M., Kim, S., Nechushtan, H. & Razin, E. (2009). *Mol. Cell*, **34**, 603–611. 10.1016/j.molcel.2009.05.019 19524539

