# Supplementary material for: The DRS–AIMP2–EPRS subcomplex acts as a pivot in the multi-tRNA synthetase complex
Source: IUCrJ. 2019 Aug 24;6(Pt 5):958–67. doi: 10.1107/S2052252519010790 (PMC6760448; doi:10.1107/S2052252519010790)
Supplement: Supplementary file 1 [file m-06-00958-sup1.pdf]

IUCrJ

Volume 6 (2019)

Supporting information for article:

The DRS–AIMP2–EPRS subcomplex acts as a pivot in the multi-tRNA synthetase complex

Hyunggu Hahn, Sang Ho Park, Hyun-Jung Kim, Sunghoon Kim and Byung Woo Han

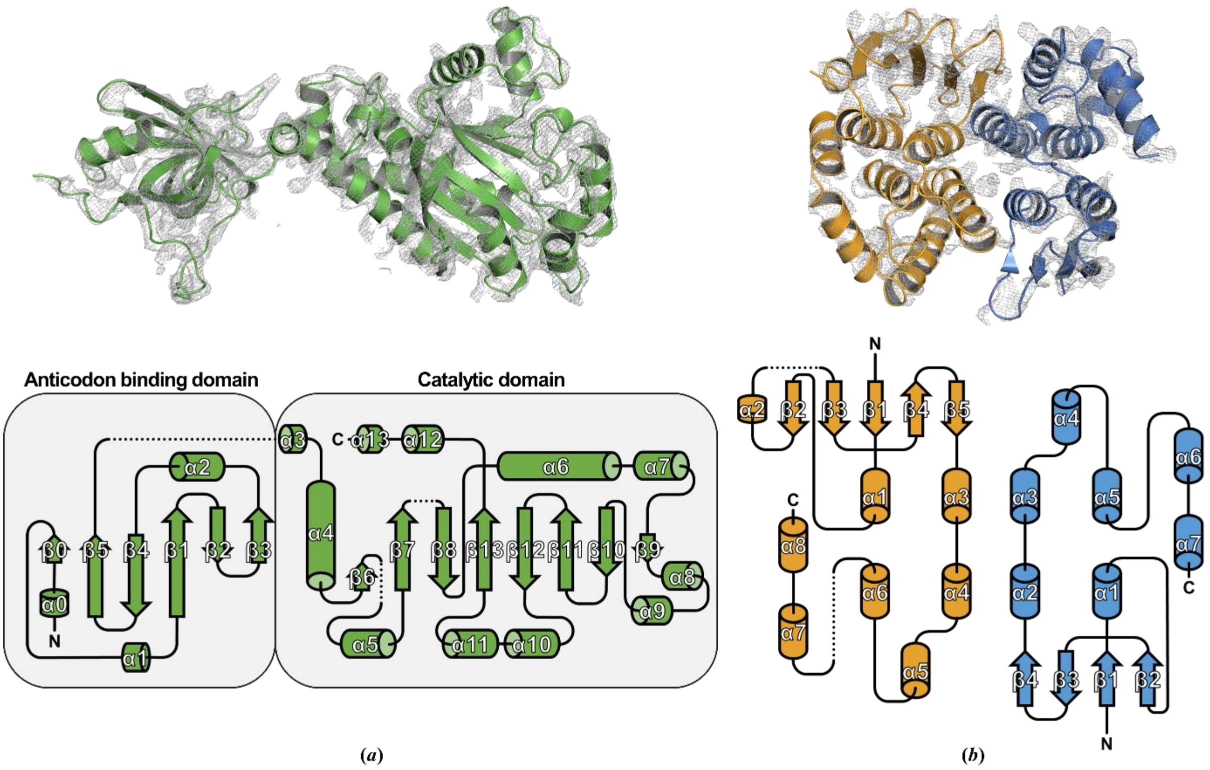

Figure S1 Electron density map and topology diagram of DRS and heterodimeric AIMP2$_{GST}$-EPRS$_{GST}$ structures. (*a*) Individual structure with 2Fo-Fc electron density map contoured at 1.5 σ represented in gray mesh (top) and corresponding topology diagram (bottom) of DRS. Two set of β-sheets each in the anticodon-binding and the catalytic domains are illustrated with surrounding α-helices showing a typical fold of Class IIa ARS domains. The flexible loop regions in β5-α3, β6-α5, and β7-β8, of which structures could not be modeled, are represented as dashed lines. (*b*) Individual structures with 2Fo-Fc electron density map contoured at 1.5 σ represented in gray mesh (top) and corresponding topology diagram (bottom) of heterodimeric AIMP2$_{GST}$-EPRS$_{GST}$. Both proteins show a typical GST domain fold with an N-terminal sub-domain consisting of a β-sheet and a C-terminal sub-domain consisting of an α-helix bundle. AIMP2 contains an extra helix-loop-strand motif (β2-α2) after first helix α1, comprising a five-stranded β-sheet in the N-terminal sub-domain. The interface between AIMP2GST and EPRS$_{GST}$ consists of α3 and α4 of AIMP2$_{GST}$ and α2 and α3 of EPRS$_{GST}$. The flexible loop regions in α2-β3, of which structures could not be modeled, are represented as dashed lines.

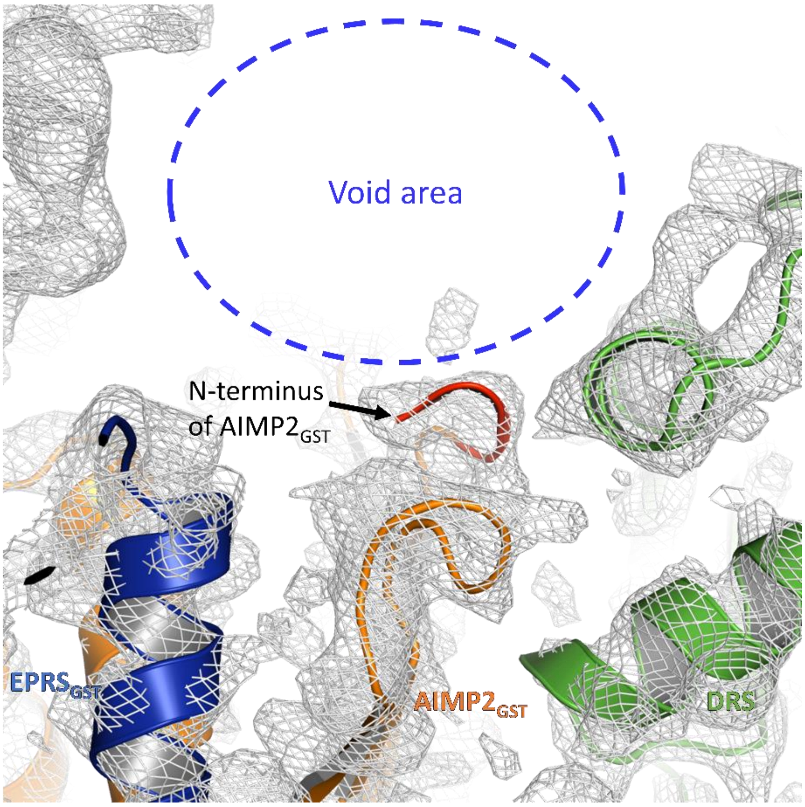

Figure S2 N-terminal region of $AIMP2_{GST}$ in the DA2E sub-complex. 2*Fo*-*Fc* electron density map contoured at 1.5 σ in gray mesh around the N-terminus of $AIMP2_{GST}$ reveals a void area free of any residual electron density. The N-terminal region of AIMP2-DX2-S34 (residues 34−45, 115−116) was too flexible to be modeled. DRS, $AIMP2_{GST}$, and $EPRS_{GST}$ are represented by green, orange, and blue cartoons, respectively. The N-terminus of $AIMP2_{GST}$ is shown in red cartoon. The void area is marked with a blue dashed circle.

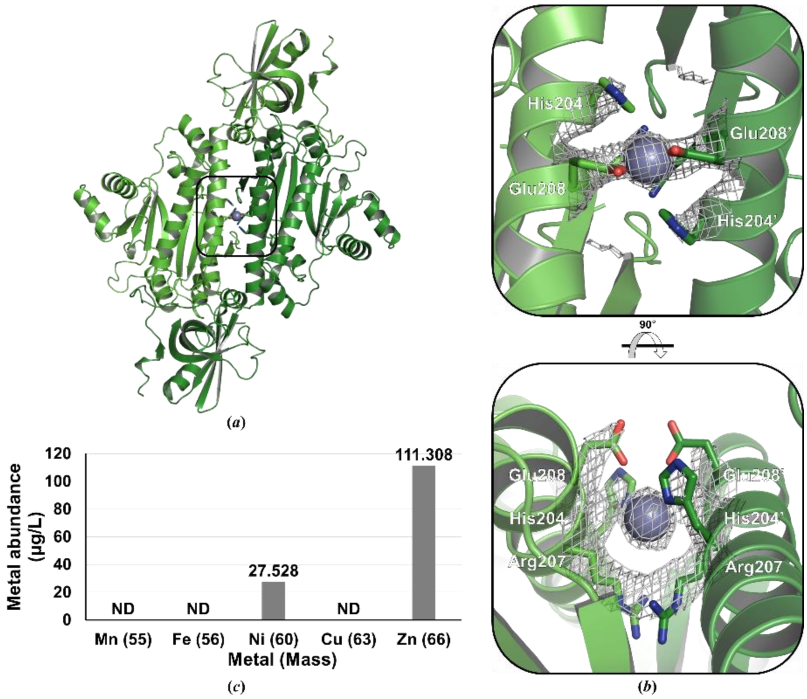

Figure S3 Identification of metal ions found at the DRS dimeric interfaces. (*a*) Overall view of the modeled zinc ion at the DRS dimeric interface. Residues from each monomer contribute to coordinating the zinc atom (gray). (*b*) Close-up view of interactions between DRS molecules and the modeled zinc ion. Residues His204 and Glu208 from each DRS monomer coordinate the zinc ion. Adjacent Arg207 residues do not seem to directly contribute to metal binding at the interface. 2*Fo*-*Fc* electron density map contoured at 1.5 σ around the zinc ion (gray mesh) shows the sidechain arrangement of nearby amino acid residues. (*c*) Metal content analysis results of the purified DA2E sub-complex by the Inductively Coupled Plasma – Mass Spectrometry (ICP-MS). Among five metal ions screened for identification, only nickel and zinc ions were detected at 27.528 μg/L and 111.308 μg/L, respectively (ND: not detected).

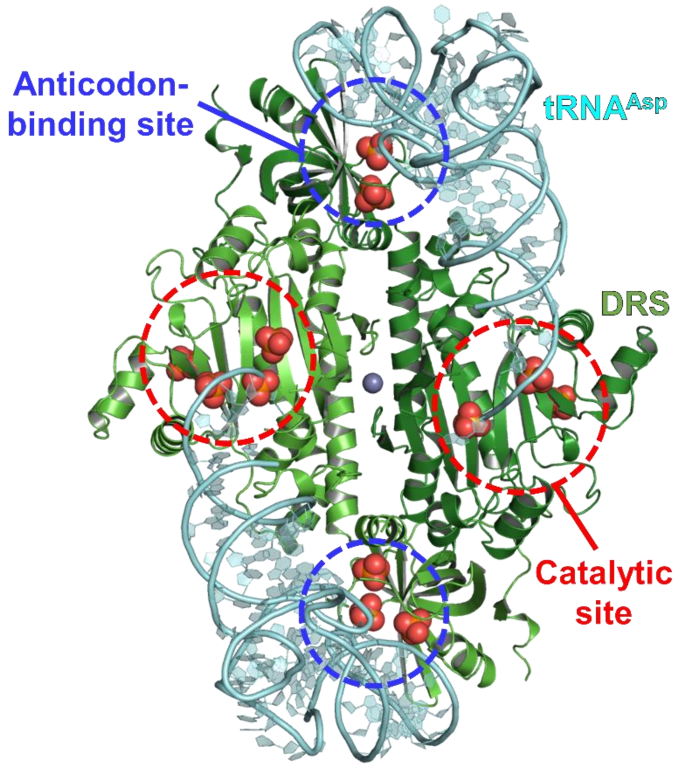

Figure S4 Phosphate ions at the putative $tRNA^{Asp}$ binding sites of DRS molecules. Extra electron densities observed at the $tRNA^{Asp}$ binding sites of DRS molecules (catalytic sites and anticodon-binding sites) were modeled as phosphate ions present in the crystallization solution at high concentrations. Bound phosphate ions seem to mimic phosphate moieties of the $tRNA^{Asp}$ backbone. $tRNA^{Asp}$ was modeled onto our DRS structure based on the yeast DRS-$tRNA^{Asp}$ complex structure (PDB ID: 1ASY). DRS and $tRNA^{Asp}$ are represented by green and cyan cartoons, respectively. Phosphate ions and a zinc ion are shown by red and gray spheres, respectively. Catalytic sites and anticodon-binding sites are marked with red and blue dashed circles, respectively.
